# Interaction between HER2 and ATM predicts poor survival in bladder cancer patients

**DOI:** 10.1111/jcmm.17512

**Published:** 2022-09-03

**Authors:** Nada Albarakati, Alaa Al‐Shareeda, Majed Ramadan, Batla Al‐Sowayan, Ola Negm, Taoufik Nedjadi

**Affiliations:** ^1^ Department of Cellular Therapy and Cancer Research King Abdullah International Medical Research Center Jeddah Saudi Arabia; ^2^ King Saud bin Abdulaziz University for Health Sciences, Ministry of the National Guard – Health Affairs Riyadh Saudi Arabia; ^3^ Department of Cellular Therapy and Cancer Research King Abdullah International Medical Research Center Riyadh Saudi Arabia; ^4^ Department of the Saudi Biobank King Abdullah International Medical Research Center Riyadh Saudi Arabia; ^5^ Department of Population Health Research King Abdullah International Medical Research Center Jeddah Saudi Arabia; ^6^ School of Medicine University of Nottingham Nottingham UK; ^7^ Microbiology and Immunology Department, Faculty of Medicine Mansoura University Mansoura Egypt

**Keywords:** ATM, Bladder cancer, HER2, Prognosis

## Abstract

Human Epidermal Growth Factor Receptor 2 (HER2) overexpression is considered one of the interesting prognostic biomarkers in bladder cancer. However, the mechanism of bladder cancer development in relation to HER2 status remains to be elucidated. In this study, we investigated HER2‐Ataxia telangiectasia mutated (ATM) kinase interaction and their impact on patient survival and cancer aggressiveness. Using the Cancer Genome Atlas (TCGA) cohorts, we demonstrated that ATM expression (protein/mRNA) is increased in HER2 deficient compared with proficient HER2 patients. This finding was then validated using the Gene Expression Omnibus database (GEO). Correlation analysis (using low expression vs high expression as a discriminator) revealed a significant association of ATM low and HER2 high status with several clinicopathological variables such as high tumour grade, late disease stage and tumour shape. Kaplan–Meier survival analysis indicated that ATM low and HER2 high is a powerful prognosticator of both overall survival (OS) and disease‐free survival (DFS). Furthermore, using bioinformatics and protein/protein interaction analyses, we identified 66 putative overlapping proteins with direct link between HER2 and ATM most of which are functionally involved in transcription regulation, apoptotic process and cell proliferation. Interestingly, the results showed that these proteins are strongly linked with PI3K‐Akt pathway, p53 pathway and microRNAs in cancer. Altogether, our data pinpoint an important biological role of the interconnection between HER2 and ATM. The latter appear to be an independent prognostic biomarker and may serve as targets to develop novel combination therapies to improve the outcome of patients with bladder cancer.

## 1 INTRODUCTION

Bladder cancer is the 10th most diagnosed type of cancer globally. In men, it is the 4th most common cancer and the 8th leading cause of cancer death.^1^, ^2^ Based on the latest Globocan data, approximately 580,000 new cases and 220,000 deaths due to bladder cancer occurred in 2020 and are expected to double in the upcoming years.^2^ Urothelial bladder carcinoma is the most common type of the disease as it originates mainly from the inner layer of the bladder (urothelium). Approximately 75% of urothelial cancer cases are classified as non‐muscle invasive bladder cancer (NMIBC) with the remaining 25% being muscle invasive bladder cancer (MIBC) which is likely to metastasize to lymph nodes or other organs.^3^ Despite advances in cancer diagnosis and therapy, bladder cancer remains a source of challenge to clinicians and healthcare providers due to high recurrence rates and the aggressive phenotype.^4^ Hence, prognostic biomarkers are desperately needed to predict outcome and optimize the treatment protocol for bladder cancer patients. Recent advances in sequencing and genomics have yielded a wealth of information that could be used in personalized medicine and targeted cancer therapy. For instance, Human Epidermal Growth Factor Receptor 2 (HER2), Fibroblast Growth Factor Receptor (FGFR), Mammalian target of rapamycin (mTOR) and immune checkpoint inhibitors; currently used in clinical practice.^5^, ^6^, ^7^, ^8^, ^9^, ^10^ Several studies provide evidence on the prognosis benefit of HER2 amplification or overexpression levels in bladder cancer, suggesting the potential benefits of HER2 targeted therapies on patients' survival.^11^, ^12^, ^13^, ^14^

HER2 is a transmembrane glycoprotein receptor tyrosine kinase of the EGFR (growth factor receptor family). Increased activity of HER2 has been evaluated and was associated with poor prognostic in breast, gastric and bladder cancers.^15^ Recently, 2411 bladder tumours were sequenced and six distinct molecular subtypes were identified; HER2‐like is one of them.^16^, ^17^ Today, HER2 is considered one of the important prognostic biomarkers in bladder cancer.^18^, ^19^ Early data revealed that HER2‐targeted therapy is beneficial for metastatic or advanced carcinoma patients with HER2 overexpression.^20^, ^21^ However, a phase II trial for patients with advanced or metastatic urothelial cancer overexpressing HER2 treated with trastuzumab combined with chemotherapy showed similar results achieved with chemo alone.^22^, ^23^ A more recent study indicated that patients with recurrent urothelial bladder cancer and amplified *HER2* gene benefited from trastuzumab and chemotherapy.^23^ The aforementioned studies highlight the clinical relevance of HER2 and the utility of anti‐HER2 targeted therapy as an alternative treatment in bladder cancer. However, the exact molecular mechanisms underlying the effect of HER2, including the crosstalk between HER2 and other signalling pathways such as Ataxia telangiectasia mutated (ATM) kinase, remain to be elucidated.^24^ ATM is a tumour suppressor gene that works as a genomic stability guardian due to its essential role in DNA damage response and repair.^25^ In bladder cancer, *ATM*/*RB1* mutations predicted poorer survival.^26^, ^27^ In breast cancer, ATM activity reduced recurrence time in patients with invasive HER2‐positive; moreover ATM was found to be involved in HER2 tumour progression.^28^, ^29^ In gastric cancer, ATM low protein expression subtype was exclusive with HER2 high protein expression.^30^

The aim of the current study was to describe an integrative analysis of HER2 and ATM interaction using the Cancer Genome Atlas (TCGA) bladder cancer cohorts to highlight the importance of both genes as potential prognosticators for bladder cancer patients. We sought to determine the expression of HER2 and ATM at the protein and mRNA levels in bladder cancer cohorts to understand their relationship and investigate their impact on patient survival and cancer aggressiveness. Also, we attempted to identify the overlapping proteins between HER2 and ATM pathways to provide a deeper insight into the molecular interactions and functional mechanisms between these two biomarkers.

## 2 MATERIALS AND METHODS

### 2.1 Study cohorts

The current study is a retrospective study using four cohorts. *Cohort one*; *ERBB2* and *ATM* mRNA expression in a panel of different cancer types (bladder, breast, colon, kidney renal clear cell, kidney renal papillary cell, kidney chromophobe, uterine corpus endometrial, thyroid, liver and stomach) extracted from TCGA datasets along with normal match. Excluding cancer types with less than 19 samples and cancer types with no significant different between the *mRNA* levels in tumours and the respective normal tissues. Data were examined in UALCAN a publicly available interactive online portal (http://ualcan.path.uab.edu/index.html).^31^ *Cohort two*; TCGA MIBC dataset (*n* = 413) was used to evaluate HER2 and ATM mRNA, protein expressions and clinicopathological information provided by cBioPortal.^32^, ^33^ In this cohort, mRNA expression z‐scores (RNA Seq V2 RSEM) measured by Agilent microarray and protein expression z‐scores measured by Reverse Phase Protein Array (RPPA). All data extracted from cBioPortal (https://www.cbioportal.org/) originally from Bladder Cancer (TCGA, Cell 2017)^34^ and can be found in Table S1. We defined mRNA and protein under‐ or over‐expression if the value is greater/less than the median cut‐point of HER2 factor. *Cohort three*; From the Gene Expression Omnibus (GEO) database (https://www.ncbi.nlm.nih.gov/gds/), the GSE13507 (Platform GPL6102) dataset was obtained.^35^, ^36^ In this cohort, 10 normal bladder mucosae samples, 58 normal looking bladder mucosae surrounding cancer and 165 primary bladder cancer samples were profiled for *ERBB2* and *ATM* mRNA expression using Illumina human‐6 v2.0 expression beadchip. *Cohort four*; GSE32548 (Platform GPL6947) also from GEO database. This cohort is 131 primary bladder cancer tumour samples analysed with Illumina HumanHT‐12 V3.0 expression beadchip.^37^

### 2.2 Data processing, Protein–Protein Interaction (PPI) network construction and co‐expressed proteins identification

In order to obtain a PPI network between HER2 and ATM, we first used Reactome (https://reactome.org/) a pathway database,^38^ to find all related pathways/functions where both of our protein targets are involved. The median score of each target from Cohort two RPPA data was used. To find both functional and physical networks between our targets, we proceed with STRING (version 11.0), multiple proteins database, which will evaluate the interactive relationships (https://string‐db.org/).^39^ Using experiments, co‐expression and co‐occurrence as active interaction sources at high confidence (0.700). All nodes with direct interaction with both HER2 and ATM were obtained, then all co‐expressed proteins were visualized by Cytoscape (https://cytoscape.org/).^40^ To analyse the network, NetworkAnalyzer,^41^ a plugin in Cytoscape, was applied to calculate the topology parameters. Then a centrality calculation was performed by Cytohubba,^42^ a plugin in Cytoscape, using degree as a topological method in order to explore the important nodes in our sub‐network.

### 2.3 Functional and pathway enrichment analysis

The Database for Annotation, Visualization and Integrated Discovery tool (DAVID; version 6.8: https://david.ncifcrf.gov/home.jsp) was used to provide Gene Ontology analysis including biological process, molecular function, cellular component and also Kyoto Encyclopedia of Gene and Genomes pathway analysis (KEGG).^43^ Pathway enrichment analysis was performed with the threshold of *p* < 0.05.

### 2.4 Statistical analyses

Data analysis was performed using JMP Pro 15 (SAS Institute Inc., USA). In the univariate analysis, Chi‐square test (*χ* ^2^) for more than five subjects per cell and Fisher exact test for less than five subjects per cell were used to evaluate the relationship between HER2 and ATM factors expression and clinicopathological variables. For the prognostic significance survival curves, Kaplan–Meier analysis was used with log‐rank comparison test. In multivariate analysis, to emphasize on HER2‐ATM interaction, Cox proportional hazard model was used for the multivariate survival analysis including all potential confounder factors. The proportional hazards assumption was checked, the relationship between log cumulative hazard and a covariate was linear. Where appropriate, two‐tailed Student's t‐test was performed using GraphPad Prism (version 8.4.3, USA). All differences were considered statistically significant at *p* < 0.05, *p* values were two‐sided; all confidence intervals were at 95%.

## 3 RESULTS

### 3.1 Analysis of HER2 and ATM expression levels in human cancers

**FIGURE 1:**
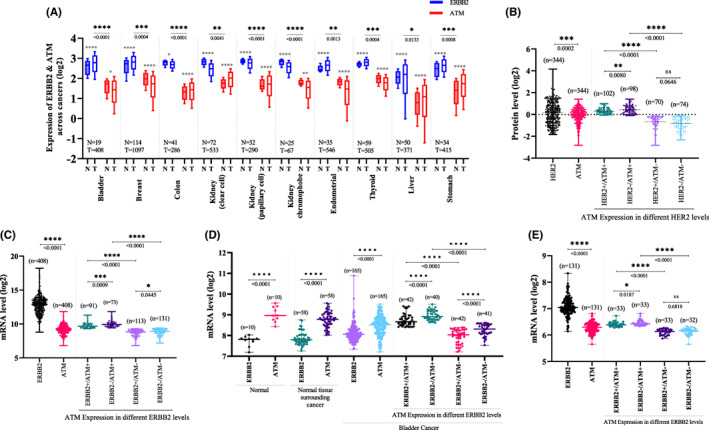
(A) Boxplot of the mRNA expression levels of *ERBB2* and *ATM* in different cancer types, along with matching normal tissue (N = normal and T = tumour). (B) Dot plot showing the protein expression levels of HER2 and ATM in bladder cancer patients, and ATM expression under different HER2 levels. (C) TCGA Dot plot showing the mRNA expression levels of *ERBB2* and *ATM* in bladder cancer patients, and *ATM* expression under different *ERBB2* levels. (D) GEO‐GSE13507 Dot plot showing the mRNA expression levels of *ERBB2* and *ATM* in bladder cancer patients, along with normal samples and *ATM* expression under different *ERBB2* levels. (E) GEO‐GSE32548 Dot plot showing the mRNA expression levels of *ERBB2* and *ATM* in bladder cancer patients, and *ATM* expression under different *ERBB2* levels. *p < 0.05, **p < 0.01, ***p < 0.001 and ****p < 0.0001. All data were analysed by the two‐tailed Student's t‐test

We initially profiled the expression pattern of *ERBB2* and *ATM* mRNA levels in a panel of different normal and tumour tissues with bioinformatics analyses using the TCGA database (*Cohort one*). The data revealed an inverse relationship between the mRNA levels in tumours and the respective normal tissues in all organs. Interestingly, we noticed that when the *ERBB2* level is high in cancer compared with the matching normal tissue, the *ATM* level in cancer shows deregulation compared with *ATM* levels in normal matched tissue. This is more obvious in bladder, breast, uterine corpus endometrial and thyroid. However, when the *ERBB2* expression in cancer is impaired compared with the matching normal tissue, the *ATM* level increases compared with the matching normal tissue, as illustrated in colon, kidney renal clear cell and kidney renal papillary cell cancers. *ERBB2* and *ATM* levels both increases in liver cancer, stomach cancer and both decreases in kidney chromophobe cancer (Figure 1A). It is also important to emphasize that the thresholds of *ERBB2* expression are higher than the thresholds of *ATM* expression in all tissues.

### 3.2 Relationship between HER2 and ATM in bladder cancer

We investigated the balancing mechanism between HER2 and ATM at protein and mRNA levels using bladder cancer TCGA cohort from the cBioPortal database. The current bladder cancer TCGA cohort (*Cohort two*) included 413 patients diagnosed with MIBC. The mean age at diagnosis was 68 years, ranging from 34 to 90 years old with a median age of 69; the median follow‐up time is 17.61 months (ranging from 0 to 165.9 months). The distribution of the clinicopathological characteristics of the patients is presented in Table 1. In this cohort, first, we found that HER2 protein expression, measured by RPPA, was significantly higher than ATM protein expression as expected; *p* = 0.0002 (Figure 1B). Interestingly, when we sub grouped ATM‐positive and ATM‐negative patients according to HER2 status, we found that ATM expression levels increased significantly in HER2 deficient patients compared with proficient HER2 patients (*p* = 0.008).

**TABLE 1:** Association of HER2, ERBB2 expressions and clinicopathological variables in TCGA dataset

				Whole cohort					Whole cohort				
		Whole cohort		*ERBB2* non‐amplified		*ERBB2* amplified			HER2 Low expression		HER2 High expression		
		*N*	%	*N*	%	*N*	%	*p* value	*N*	%	*N*	%	*p* value
Group age (Years)	>60	392	94.92%	191	46.25%	197	47.70%	0.347	159	38.50%	166	40.19%	0.143
	≤60	20	4.84%	12	2.91%	8	1.94%		12	2.91%	6	1.45%	
	Unknown	1	0.24%	1	0.24%	0	0.00%		1	0.24%	0	0.00%	
Gender	Male	304	73.61%	144	34.87%	157	38.01%	0.195	125	30.27%	133	32.20%	0.365
	Female	108	26.15%	59	14.29%	48	11.62%		46	11.14%	39	9.44%	
	Unknown	1	0.24%	1	0.24%	0	0.00%		1	0.24%	0	0.00%	
Tumour grade	Low Grade	21	5.08%	199	48.18%	185	44.79%	**0.003**	170	41.16%	152	36.80%	**<0.001**
	High Grade	388	93.95%	4	0.97%	17	4.12%		0	0.00%	19	4.60%	
	Unknown	4	0.97%	1	0.24%	3	0.73%		2	0.48%	1	0.24%	
Tumour stage	T3b	82	19.85%	47	11.38%	34	8.23%	0.161	44	10.65%	29	7.02%	**0.001**
	T3a	71	17.19%	34	8.23%	37	8.96%		35	8.47%	22	5.33%	
	T2b	56	13.56%	32	7.75%	24	5.81%		24	5.81%	21	5.08%	
	T4a	43	10.41%	19	4.60%	24	5.81%		15	3.63%	19	4.60%	
	T3	42	10.17%	21	5.08%	21	5.08%		20	4.84%	18	4.36%	
	T2	38	9.20%	14	3.39%	24	5.81%		4	0.97%	25	6.05%	
	Unknown	33	7.99%	18	4.36%	15	3.63%		19	4.60%	8	1.94%	
	T2a	27	6.54%	7	1.69%	18	4.36%		6	1.45%	17	4.12%	
	T4	11	2.66%	7	1.69%	3	0.73%		4	0.97%	6	1.45%	
	T4b	5	1.21%	2	0.48%	3	0.73%		1	0.24%	3	0.73%	
	T1	3	0.73%	2	0.48%	1	0.24%		0	0.00%	2	0.48%	
	T0	1	0.24%	1	0.24%	0	0.00%		0	0.00%	1	0.24%	
	TX	1	0.24%	0	0.00%	1	0.24%		0	0.00%	1	0.24%	
Disease stage	Stage III	141	34.14%	85	20.58%	55	13.32%	**0.015**	73	17.68%	46	11.14%	**0.012**
	Stage IV	136	32.93%	57	13.80%	77	18.64%		53	12.83%	62	15.01%	
	Stage II	131	31.72%	59	14.29%	71	17.19%		45	10.90%	60	14.53%	
	Unknown	3	0.73%	2	0.48%	1	0.24%		1	0.24%	2	0.48%	
	Stage I	2	0.48%	1	0.24%	1	0.24%		0	0.00%	2	0.48%	
Tumour shape	Non‐papillary	274	66.34%	151	36.56%	119	28.81%	**<0.001**	135	32.69%	93	22.52%	**<0.001**
	Papillary	133	32.20%	48	11.62%	85	20.58%		34	8.23%	77	18.64%	
	Unknown	6	1.45%	5	1.21%	1	0.24%		3	0.73%	2	0.48%	
Lymph node	YES	296	71.67%	149	36.08%	143	34.62%	0.144	133	32.20%	117	28.33%	**0.036**
	NO	79	19.13%	33	7.99%	46	11.14%		26	6.30%	41	9.93%	
	Unknown	38	9.20%	22	5.33%	16	3.87%		13	3.15%	14	3.39%	
Lymph node stage	N0	239	57.87%	133	32.20%	104	25.18%	**0.021**	104	25.18%	97	23.49%	**0.036**
	N2	76	18.40%	27	6.54%	48	11.62%		23	5.57%	43	10.41%	
	N1	47	11.38%	21	5.08%	25	6.05%		25	6.05%	13	3.15%	
	NX	36	8.72%	14	3.39%	22	5.33%		13	3.15%	14	3.39%	
	N3	8	1.94%	4	0.97%	4	0.97%		3	0.73%	4	0.97%	
	Unknown	7	1.69%	5	1.21%	2	0.48%		4	0.97%	1	0.24%	
Metastasis	YES	213	51.57%	114	27.60%	96	23.24%	0.052	105	25.42%	73	17.68%	**<0.001**
	NO	196	47.46%	87	21.07%	108	26.15%		63	15.25%	99	23.97%	
	Unknown	4	0.97%	3	0.73%	1	0.24%		4	0.97%	0	0.00%	
Metastasis stage	MX	202	48.91%	108	26.15%	91	22.03%	0.151	103	24.94%	65	15.74%	**<0.001**
	M0	196	47.46%	87	21.07%	108	26.15%		63	15.25%	99	23.97%	
	M1	11	2.66%	6	1.45%	5	1.21%		2	0.48%	8	1.94%	
	Unknown	4	0.97%	3	0.73%	1	0.24%		4	0.97%	0	0.00%	
Family history of cancer	NO	262	63.44%	119	28.81%	142	34.38%	**0.020**	104	25.18%	117	28.33%	0.104
	YES	147	35.59%	83	20.10%	61	14.77%		67	16.22%	52	12.59%	
	Unknown	4	0.97%	2	0.48%	2	0.48%		1	0.24%	3	0.73%	
Smoking	YES	259	62.71%	134	32.45%	123	29.78%	0.234	109	26.39%	101	24.46%	0.376
	NO	154	37.29%	70	16.95%	82	19.85%		63	15.25%	71	17.19%	

*Note*: *N* number of cases; significant values (*p* < 0.05) are highlighted in bold.

Similarly, at mRNA levels (Figure 1C), total *ERBB2* was significantly higher than *ATM* levels (*p* < 0.0001), and high *ATM* expression was observed when *ERBB2* was low compared with high *ERBB2* (*p* = 0.0009). This data was validated in an independent cohort using GEO GSE13507 dataset accessible from the National Center for Biotechnology Information (NCBI).^35^, ^36^ In this cohort (*Cohort three*), 10 normal bladder mucosae samples, 58 normal looking bladder mucosae surrounding cancer and 165 primary bladder cancer samples were profiled for *ERBB2* and *ATM* mRNA expression using Illumina human‐6 v2.0 expression beadchip. Figure 1D, shows total *ERBB2* expression (mean = 7.8) significantly low than *ATM* (mean = 9) in both normal sets (*p* < 0.0001). However, in primary cancer samples, *ERBB2* mean increase to 8.1 with a decrease of *ATM* to 8.4 (*p* < 0.0001). Interestingly, the *ATM* levels increased significantly in *ERBB2* deficient patients compared with *ERBB2* proficient patients (*p* < 0.0001). We validated this finding with second dataset from GEO (*Cohort four*) GSE32548 (Platform GPL6947). This cohort consist of 131 primary bladder cancer tumour samples analysed with Illumina HumanHT‐12 V3.0 expression beadchip. As expected (Figure 1E), total *ERBB2* was significantly higher than *ATM* levels (*p* < 0.0001) and *ATM* levels increased significantly in *ERBB2*− compared to *ERBB2+* patients (*p* = 0.0187).

### 3.3 Relationships between ATM, HER2 factors and clinical outcome

Considering the inverse relationship between the HER2 level and the ATM level, we sought to investigate whether HER2 and ATM expression factors affects patient prognosis. To do so, first we looked at the TCGA cohort (*Cohort two*) and observed a significant impact of combining high *ERBB2*/HER2 expression on the overall survival (OS); *p* = 0.024 and disease‐free survival (DFS); with borderline significance (*p* = 0.068) (Figure S1A,B). Whereas the *ATM*/ATM expression levels had no prognostic value (Figure S1C,D). Though neither HER2 nor ATM factors alone show any significance on the OS or DFS at the protein level (Figure S2A–D) or the mRNA levels in this cohort. However, the *ERBB2* showed a significant poor DFS for patients with amplified *ERBB2*; *p* = 0.032 (Figure S3A–D). Based on Kaplan–Meier, patients with low ATM protein level and low *ATM* mRNA showed a tendency toward poor OS (*p* = 0.057), and poor DFS (*p* = 0.078) (Figure S2C,D).

**FIGURE 2:**
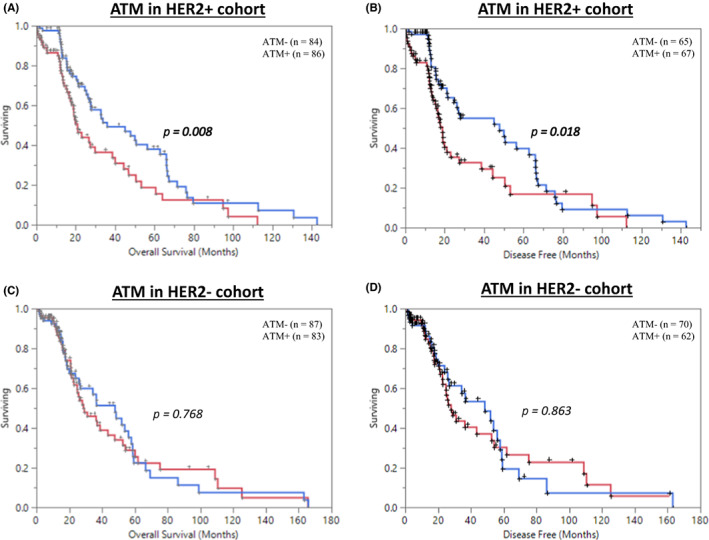
Kaplan–Meier analysis for bladder cancer data; (A) overall survival of ATM protein expression in high HER2 patients (total HER2+ *n* = 172), (B) Disease‐free survival of ATM protein expression in high HER2 patients (total HER2+ *n* = 172), (C) overall survival of ATM protein expression in low HER2 patients (total HER2− *n* = 172), (D) Disease‐free survival of ATM protein expression in low HER2 patients (total HER2− *n* = 172)

Then, we stratified patients into two subgroups according to the HER2 status. Our data indicated that patients with low ATM and high HER2 expression strongly predicts poor OS and DFS; *p* = 0.008 and *p* = 0.018, respectively (Figure 2A,B). In contrast, ATM levels did not show any difference on patients' survival when HER2 was low (Figure 2C,D). Also, no significance was seen for *ATM* mRNA levels with different *ERBB2* status (Figure S4A–D). The combination of low HER2 and high ATM expression was significantly associated with late disease stage (*p* = 0.026), no association was observed with any other clinicopathological variables (Table S2).

### 3.4 HER2‐ATM Co‐expression in bladder cancer

Interestingly, when stratified the whole population based on both HER2/ATM protein expression status, patients with low ATM/high HER2 appear to have significant poor outcome compared with the other three subgroups (ATM low/HER2 low, ATM high/HER2 high and ATM high/HER2 low) in OS (*p* = 0.025) and DFS (*p* = 0.024) (Figure S5A,B). Similarly, at the mRNA level, patients with low *ATM* and amplified *ERBB2* showed a significant poor DFS (*p* = 0.046) but not OS (Figure S5C,D).

### 3.5 Relationship between ATM/HER2 and clinicopathological features

To evaluate the relationship of ATM/HER2 status and the clinicopathological parameters, we used univariate analysis. Our data indicate that mRNA expression of *ATM*/*ERBB2* was significantly associated with tumour grade (*p* = 0.011), disease stage (*p* = 0.009) and tumour shape (*p* = 0.001). Similarly, it was identified in the ATM/HER2 protein level with tumour grade (*p* < 0.001), disease stage (*p* = 0.008), tumour shape (*p* < 0.001), in addition to lymph node (*p* = 0.046) and metastasis (*p* = 0.001) (Table 2). Multivariate analysis was conducted to investigate whether HER2/ATM expression is an independent prognostic factor. As shown in Table 3, multivariate analyses including other validated prognostic factors (such as tumour grade, lymph node, tumour shape, metastasis stage) and ATM expression (OS; *p* = 0.003, Hazard ratio = 0.343, 95% CI = 0.171–0.689, DFS; *p* = 0.003, Hazard ratio = 0.298, 95% CI = 0.134–0.667) independently predicted poor clinical outcome. HER2/ATM co‐expression was an independent prognostic of worse survivals (OS; *p* = 0.038, Hazard ratio = 2.739, 95% CI = 1.063–7.057, DFS; *p* = 0.041, Hazard ratio = 1.477; 95% CI = 1.017–2.133). Interestingly, HER2+/ATM expression group was in independent prognostic of worse survival (OS; *p* = 0.012, Hazard ratio = 0.593, 95% CI = 0.394–0.892, DFS; *p* = 0.001, Hazard ratio = 0.125; 95% CI = 0.036–0.607) but not HER2−/ATM group.

**TABLE 2:** Association of ATM/HER2 factors co‐expressions and clinicopathologic variables

		*ATM*/*ERBB2* (Gene level)									ATM /HER2 (Protein level)								
		*ATM‐/ERBB2‐*		*ATM‐/ERBB2+*		*ATM+/ERBB2+*		*ATM+/ERBB2‐*			ATM‐/HER2‐		ATM‐/HER2+		ATM+/HER2+		ATM+/HER2‐		
		*N*	%	*N*	%	*N*	%	*N*	%	*p* value	*N*	%	*N*	%	*N*	%	*N*	%	*p* value
Group age (Years)	Unknown	0	0.00%	0	0.00%	0	0.00%	1	0.24%	0.398	0	0.00%	1	0.24%	0	0.00%	0	0.00%	0.090
	≤60	7	1.69%	6	1.45%	2	0.48%	5	1.21%		9	2.18%	3	0.73%	4	0.97%	2	0.48%	
	>60	93	22.52%	98	23.73%	99	23.97%	98	23.73%		78	18.89%	82	19.85%	84	20.34%	81	19.61%	
Gender	Unknown	0	0.00%	0	0.00%	0	0.00%	1	0.24%	0.512	0	0.00%	1	0.24%	0	0.00%	0	0.00%	0.357
	Male	73	17.68%	81	19.61%	76	18.40%	71	17.19%		67	16.22%	58	14.04%	67	16.22%	66	15.98%	
	Female	27	6.54%	23	5.57%	25	6.05%	32	7.75%		20	4.84%	27	6.54%	21	5.08%	17	4.12%	
Tumour grade	Unknown	0	0.00%	1	0.24%	2	0.48%	1	0.24%	**0.011**	0	0.00%	2	0.48%	0	0.00%	1	0.24%	**<0.001**
	low grade	1	0.24%	11	2.66%	6	1.45%	3	0.73%		0	0.00%	0	0.00%	10	2.42%	9	2.18%	
	high grade	99	23.97%	92	22.28%	93	22.52%	100	24.21%		87	21.07%	84	20.34%	78	18.89%	73	17.68%	
Disease stage	Unknown	1	0.24%	0	0.00%	1	0.24%	1	0.24%	**0.009**	0	0.00%	1	0.24%	0	0.00%	2	0.48%	**0.008**
	Stage I	0	0.00%	0	0.00%	1	0.24%	1	0.24%		0	0.00%	0	0.00%	1	0.24%	1	0.24%	
	Stage II	36	8.72%	39	9.44%	32	7.75%	23	5.57%		30	7.26%	15	3.63%	36	8.72%	24	5.81%	
	Stage III	37	8.96%	33	7.99%	22	5.33%	48	11.62%		30	7.26%	44	10.65%	22	5.33%	23	5.57%	
	Stage IV	26	6.30%	32	7.75%	45	10.90%	31	7.51%		27	6.54%	26	6.30%	29	7.02%	33	7.99%	
Tumour shape	Unknown	2	0.48%	0	0.00%	1	0.24%	3	0.73%	**0.001**	1	0.24%	2	0.48%	1	0.24%	1	0.24%	**<0.001**
	Non‐Papillary	77	18.64%	55	13.32%	64	15.50%	74	17.92%		65	15.74%	71	17.19%	46	11.14%	46	11.14%	
	Papillary	21	5.08%	49	11.86%	36	8.72%	27	6.54%		21	5.08%	13	3.15%	41	9.93%	36	8.72%	
Lymph node	Unknown	10	2.42%	4	0.97%	12	2.91%	12	2.91%	0.331	5	1.21%	8	1.94%	4	0.97%	10	2.42%	**0.046**
	NO	19	4.60%	26	6.30%	20	4.84%	14	3.39%		17	4.12%	9	2.18%	19	4.60%	22	5.33%	
	YES	71	17.19%	74	17.92%	69	16.71%	78	18.89%		65	15.74%	69	16.71%	65	15.74%	51	12.35%	
Metastasis	Unknown	0	0.00%	0	0.00%	1	0.24%	3	0.73%	0.174	3	0.73%	1	0.24%	0	0.00%	0	0.00%	**0.001**
	NO	40	9.69%	53	12.83%	55	13.32%	47	11.38%		31	7.51%	32	7.75%	56	13.56%	43	10.41%	
	YES	60	14.53%	51	12.35%	45	10.90%	54	13.08%		53	12.83%	53	12.83%	32	7.75%	40	9.69%	

*Note*: *N* number of cases; significant values (*p* < 0.05) are highlighted in bold.

**TABLE 3:** Multivariate analysis for predictors of overall and disease‐free survival

Factors	Overall survival				Disease‐free survival			
	Hazard ratio	95% confidence interval			Hazard ratio	95% confidence interval		
		Lower bound	Upper bound	*p* value		Lower bound	Upper bound	*p* value
Gender	0.806	0.558	1.164	0.243	0.681	0.432	1.043	0.078
Tumour grade	0.431	0.218	0.851	**0.019**	0.387	0.182	0.823	**0.014**
Tumour Stage	0.713	0.356	1.431	0.341	0.862	0.652	1.133	0.288
Lymph Node	0.468	0.293	0.748	**0.002**	0.470	0.257	0.886	**0.020**
Tumour Shape	1.866	1.326	2.627	**<0.0001**	2.594	1.671	4.032	**<0.0001**
Metastasis Stage	1.249	0.882	1.769	0.209	1.635	1.077	2.509	**0.021**
ATM expression	0.343	0.171	0.689	**0.003**	0.298	0.134	0.667	**0.003**
HER2 expression	0.970	0.689	1.367	0.864	0.832	0.550	1.259	0.385
HER2/ATM expression	2.739	1.063	7.057	**0.038**	1.477	1.017	2.133	**0.041**
HER2+/ATM expression	0.593	0.394	0.892	**0.012**	0.125	0.036	0.607	**0.001**
HER2−/ATM expression	0.938	0.609	1.434	0.768	1.276	0.794	2.050	0.311

Abbreviations: ATM, Ataxia telangiectasia mutated; HER2, Human Epidermal growth factor Receptor 2; HER2/ATM, co‐expression. Hazard ratio, 95% Confidence Interval and *p* value are shown. Significant results are highlighted in bold.

### 3.6 Identification of overlapping proteins between HER2 and ATM

**FIGURE 3:**
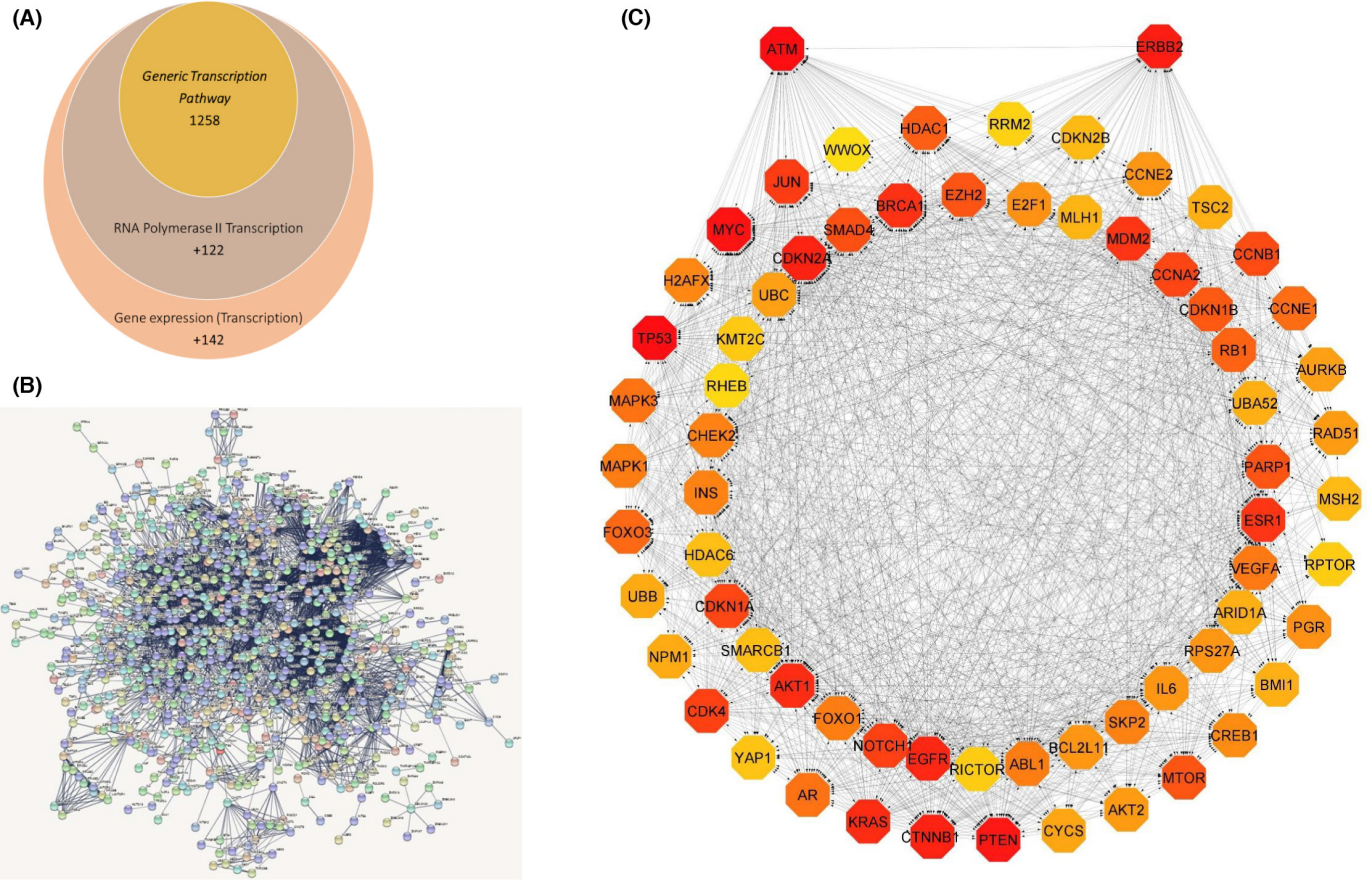
(A) Venn diagram of common pathways between HER2 and ATM. (B) Protein–protein interaction network demonstrating all overlapping proteins. (C) Protein–protein interaction of 66 proteins with direct link to both HER2 and ATM

In order to understand how the ATM/HER2 status can affect the prognosis for bladder cancer patients, we investigated the overlapping PPI between the HER2 and ATM pathways using Reactome pathway database tool. By using the median score of both proteins from the TCGA *Cohort two*, we were able to identify 217 pathways for HER2 and 140 pathways for ATM. Three pathways only shows both proteins; Generic Transcription Pathway, RNA Polymerase II Transcription and Gene expression (Transcription); Figure 3A. Next, we analysed PPI between HER2, ATM and all proteins in the three pathways by submitting all data to STRING (PPI Networks); Figure 3B. Our network with high confidence and experiments, co‐expression and co‐occurrence as active interaction sources; shows a total of 1311 nodes and 5028 edges. Among all these nodes a total of 66 overlapping proteins were directly linked between HER2 and ATM (Figure 3C) visualized manually by Cytoscape software. In addition, Networkanalyzer and Cytohubba analysis of the sub‐network shows 68 nodes and 1426 edges. Table 4 is a PPI ranked by degree method illustrating the degree, betweenness centrality and closeness centrality. Also, 11 topological analysis methods were applied (Table S3). Then, we assessed the significant differentness in the overlapping proteins in the stratified patients cohort (*Cohort two*) according to HER2‐ATM status. Data identified that in the low HER2 cohort and different ATM levels (high/low); ABL1, SMAD4, RB1 and PARP1 were significantly upregulated and AKT1, AKT2, TSC2, RPTOR and mTOR were significantly downregulated in bladder cancer (Table S4).

**TABLE 4:** The 68 PPI network ranked by degree method

Rank	Name	Uniprot ID	Degree	Betweenness centrality	Closeness centrality	Rank	Name	Uniprot ID	Degree	Betweenness centrality	Closeness centrality
1	TP53	Q8J016	67	0.0233	1.0000	35	SKP2	Q13309	40	0.0030	0.7128
1	ATM	Q13315	67	0.0233	1.0000	35	CHEK2	Q9UGF1	40	0.0034	0.7128
3	MYC	P01106	66	0.0223	0.9853	35	INS	P01308	40	0.0025	0.7128
4	PTEN	P60484	65	0.0181	0.9710	38	H2AFX	P16104	39	0.0044	0.7053
5	ERBB2	P04626	64	0.0210	0.9571	39	CREB1	P16220	37	0.0019	0.6907
6	CTNNB1	P35222	63	0.0151	0.9437	40	PGR	P06401	36	0.0012	0.6837
6	CDKN2A	P42771	63	0.0162	0.9437	40	E2F1	Q01094	36	0.0017	0.6837
8	EGFR	Q9H2C9	62	0.0165	0.9306	42	RPS27A	Q9UPK7	35	0.0017	0.6768
9	KRAS	P01116	60	0.0131	0.9054	42	BCL2L11	Q8WYM1	35	0.0007	0.6768
9	AKT1	P31749	60	0.0142	0.9054	42	IL6	P05231	35	0.0006	0.6768
11	BRCA1	P38398	58	0.0135	0.8816	42	CCNE2	O96020	35	0.0022	0.6768
12	MDM2	Q53XW0	57	0.0103	0.8701	46	RAD51	E9PI54	34	0.0033	0.6700
12	ESR1	P03372	57	0.0099	0.8701	46	AKT2	P31751	34	0.0018	0.6700
14	JUN	P05412	55	0.0101	0.8481	48	AURKB	Q96GD4	33	0.0020	0.6634
15	CDK4	P11802	54	0.0091	0.8375	48	UBC	Q9UPK7	33	0.0015	0.6634
15	NOTCH1	P46531	54	0.0090	0.8375	50	CYCS	P99999	32	0.0006	0.6569
17	CCNA2	P20248	53	0.0079	0.8272	51	NPM1	Q96DC4	31	0.0009	0.6505
17	CDKN1A	P38936	53	0.0065	0.8272	51	UBB	Q9UPK7	31	0.0011	0.6505
19	CCNB1	P14635	52	0.0082	0.8171	53	TSC2	Q8TAZ1	30	0.0012	0.6442
20	SMAD4	Q13485	51	0.0092	0.8072	53	ARID1A	Q9HBJ5	30	0.0012	0.6442
21	EZH2	Q15910	50	0.0069	0.7976	53	BMI1	P35226	30	0.0007	0.6442
21	MTOR	P42345	50	0.0064	0.7976	53	UBA52	Q9UPK7	30	0.0011	0.6442
21	PARP1	P09874	50	0.0061	0.7976	57	MLH1	P40692	29	0.0012	0.6381
24	CDKN1B	P46527	49	0.0050	0.7882	58	MSH2	P43246	28	0.0013	0.6321
25	HDAC1	Q13547	48	0.0051	0.7791	58	CDKN2B	P42772	28	0.0005	0.6321
26	RB1	P06400	47	0.0047	0.7701	60	SMARCB1	Q12824	27	0.0007	0.6262
27	FOXO3	O43524	46	0.0046	0.7614	60	HDAC6	Q9UBN7	27	0.0014	0.6262
28	CCNE1	P24864	45	0.0047	0.7528	62	YAP1	P46937	25	0.0012	0.6147
29	MAPK3	P27361	44	0.0043	0.7444	63	KMT2C	Q8NEZ4	23	0.0005	0.6036
29	AR	P10275	44	0.0028	0.7444	64	RICTOR	Q6R327	22	0.0006	0.5982
31	VEGFA	Q9H1W9	42	0.0021	0.7283	64	RPTOR	Q8N122	22	0.0003	0.5982
32	MAPK1	P28482	41	0.0030	0.7204	66	RRM2	P31350	18	0.0002	0.5776
32	ABL1	P00519	41	0.0031	0.7204	67	RHEB	Q15382	17	0.0000	0.5726
32	FOXO1	Q12778	41	0.0032	0.7204	68	WWOX	Q9NZC7	11	0.0001	0.5447

### 3.7 Functional and pathway enrichment analyses

**FIGURE 4:**
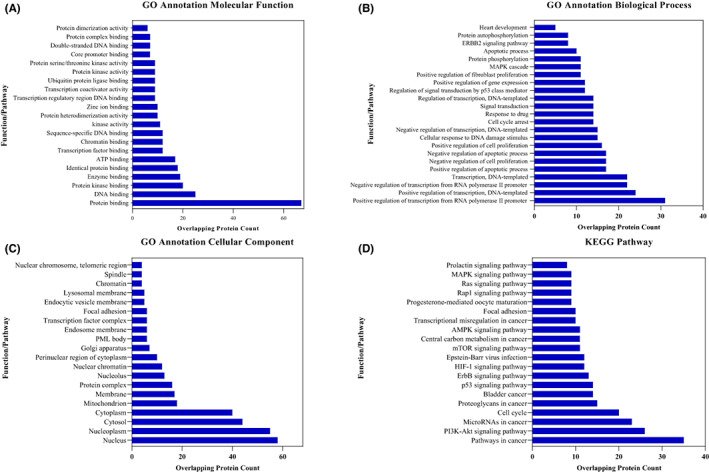
GO functional enrichment and KEGG pathway analyses of the 66 overlapping proteins, HER2 and ATM. (A) The top significant enriched GO annotation Molecular Function, (B) Biological Process, (C) Cellular Component, (D) KEGG pathway analyses

Next, we sought to analyse the functional enrichment pathways of the ATM/HER2 using the TCGA‐Bladder cancer cohort. A list of overlapping proteins was uploaded to DAVID software to identify significant GO categories and KEGG pathways. The results demonstrated that HER2/ATM overlapping proteins were markedly enriched in Molecular Function, including protein binding, DNA binding, protein kinase binding, enzyme binding and ATP binding (Figure 4A). GO Biological Process analysis showed enrichment of positive regulation of transcription from RNA polymerase II promoter, positive regulation of transcription, DNA‐templated, positive regulation of apoptotic process and negative regulation of cell proliferation (Figure 4B). Also, GO Cellular component analysis revealed that the overlapping proteins are significantly enriched in several sub‐cellular compartments including the nucleus, nucleoplasm, cytosol, cytoplasm and mitochondrion (Figure 4C). As for the results of KEGG pathway analysis, the data indicated that the overlapping proteins are mainly enriched in pathways in cancer, PI3K‐Akt signalling pathway, microRNAs in cancer, cell cycle and p53 signalling pathway (Figure 4D); fully functional and pathway enrichment analyses are shown in Figure S5.

## 4 DISCUSSION

Bladder cancer is a deadly disease characterized by high phenotypic and molecular heterogeneity. Next generation sequencing (NGS) revealed that bladder cancer possesses a high mutational burden compared with all cancers.^44^, ^45^ The process of bladder cancer development and progression involved activation of oncogenes, such as HER2, and inactivation of tumour suppressor genes, such as ATM. Many studies have reported essential roles played by HER2 and ATM in bladder carcinogenesis individually.^18^, ^46^, ^47^ Published data reported amplification of the *ERBB2* gene in up to 42% and up‐regulation of protein expression in up to 30% of bladder cancer cases. The same report indicated an inactivating mutation of *ATM* gene in 14% of bladder cancers.^48^ The dualism in the function of both genes and their prognostic value has never been investigated in bladder cancer. Our investigation examined the co‐expression of HER2‐ATM factors and assessed their prognostic and clinical significance in bladder cancer.

Using bioinformatics analyses, we observed an inversed relationship between *ERBB2* and *ATM* mRNA expressions across many TCGA‐analysed malignancies meaning that cancers expressing high *ERBB2* mRNA levels tend to have lower *ATM* expression and this is obvious in bladder, breast, uterine corpus endometrial and thyroid cancers. The other way around is true too, meaning that cancers with low *ERBB2* mRNA expression possess increased *ATM* expression, as with the colon, kidney renal clear cell and kidney renal papillary cell. The expression patterns of *ATM*/*ERBB2* mRNAs were also investigated at the protein level in the TCGA‐bladder cancer cohort. The data demonstrated significant alteration in ATM expression according to HER2 status. Our data were in agreement with another study that attempted to explore the association of immune markers in gastric cancer patients. The authors showed that a subgroup of the analysed cohort is enriched in ATM low protein expression and HER2 high protein expression.^30^ The finding that *ATM* mRNA expression increased significantly in *ERBB2* low patients compared with *ERBB2* high patients was further validated in independent GEO cohorts. This transcriptomic and proteomic‐based analysis provides evidence of a strong correlation between inhibition of HER2 expression and increased ATM expression in bladder cancer.

We observed that HER2 overexpression or/and amplified *ERBB2* of bladder tumours strongly associated with clinicopathological variables characteristic of poor prognosis, including high tumour grade, tumour stage, late disease stage, tumour shape, lymph node and metastasis in bladder cancer, our data are consistent with previous reports.^18^, ^49^ ATM expression alone did not show a strong association with any clinical features including patients' survival. However, previous studies revealed that ATM mutation was an indicator for poor overall survival in bladder cancer compared with the wild‐type.^26^, ^50^ Recent emerging data indicated that bladder cancers harbouring ATM mutations are susceptible to increased sensitivity to 29 drugs including cisplatin, IGF‐1R inhibitor and BMS‐536924. This finding also suggested great benefit for patients with ATM mutations after receiving immune checkpoint inhibitors.^47^

Investigating the protein expression of HER2 and ATM together, we found that tumours with HER2 high/ATM low had the worst OS and DFS compared with HER2 high/ATM high, HER2 low/ATM low, or high. The expression of HER2/ATM was significantly associated with tumour grade, disease stage, tumour shape, lymph node and metastasis. Furthermore, multivariate analysis indicated that HER2‐ATM expression is an independent predictor of OS and DFS. These findings were also confirmed using mRNA expression analysis, suggesting that co‐expression of HER2 with ATM factors may be potential molecular biomarkers for predicting bladder cancer prognosis and disease aggressiveness. Our data was not in agreement with Stagni et al., who reported that ATM activation and HER2 positivity predicted the worst DFS in breast cancer patients and patients displaying ATM‐p‐negative and HER2‐positive have moderate DFS suggesting that ATM sustained tumorigenicity of HER2 in breast cancer.^28^

Understanding the molecular interactions between ATM and HER2 is particularly important in the prognosis and the treatment planning of bladder cancer patients. Reddy et al. demonstrated that phosphorylation of ATM on Ser1981 is dependent on the expression of HER2 in breast cancer mouse model.^51^ A positive feedback loop between ATM and HER2 was suggested where HER2 induces phosphorylation activation of ATM. The latter supports the binding between HER2 and HSP90, enhancing HER2 expression and tumour progression.^29^ It has been suggested that ATM's role as tumour suppressor gene altered to be tumour promoter in HER2‐positive tumours.^28^, ^29^ Yan et al.’s results indicated that silencing the activity of HER2 using specific inhibitor, shRNA or using *ERBB2* mutated cells inhibit the activation of ATM and ATR signalling pathway in response to γ‐irradiation leading to G2/M cell cycle arrest.^52^ Overall, the analysis of several cohorts of patients with various malignancies revealed a strong negative correlation between the expression of HER2 and ATM at both protein and mRNA levels. Interestingly, the inverse relationship between the two proteins HER2+/ATM‐ was significantly associated with poor DFS. This finding is particularly important and showed that HER2 and ATM may serve as a prognostic biomarker. Furthermore, DFS is regarded as a way to evaluate how well the treatment works and has also become a commonly used parameter to assess the efficacy of new cancer drugs. Published data demonstrated that targeting the ATM may impact treatment efficacy and improve the outcome of cancer patients.^47^ Altogether, this data indicates that targeting patients carrying both HER2 and ATM mutations using anti‐ATM and anti‐HER2 combination therapy may provide extra benefit for cancer patients.

We next sought to investigate the overlapping proteins between HER2 and ATM pathways to provide deeper insight into the molecular mechanisms of this relation. We identified three common pathways; Generic Transcription Pathway, RNA Polymerase II Transcription and Gene expression (Transcription), which were analysed further using PPI network and module analysis. Sixty‐six overlapping proteins were identified with a direct link to both HER2 and ATM proteins, including AKT1, TP53, BRCA1, PTEN, CHEK2, ABL1, KRAS, MSH2, EGFR, CDK4, MAPK1, RAD51, mTOR, RB1, PARP1 and more. Also, PPI centrality measure of this sub‐network indicates the importance of these intermediate proteins to the interaction between HER2 and ATM.

The pathway enrichment analysis revealed that the 66 overlapping proteins involved significantly in the regulation of apoptotic process, cell proliferation, cellular response to DNA damage stimulus, cell cycle arrest, response to a drug, regulation of gene expression, protein phosphorylation for the GO Biological process term analysis. Moreover, these overlapping proteins were enriched in KEGG pathways in cancer, PI3K‐Akt signalling, microRNAs in cancer, bladder cancer, p53 signalling, *ERBB* signalling, mTOR signalling and AMPK signalling.

Interestingly, our finding showed significant alteration to AKT1, AKT2, TSC2, RPTOR and mTOR expression, which followed ATM status when HER2 was low. ABL1, SMAD4, RB1 and PARP1 showed significant alteration in the opposite direction to ATM status with HER2 low patients. A recent study suggested a new function of HER2 in recruiting AKT1, which deactivates STING signalling and suppresses antiviral and antitumor immunity.^53^ In contrast, ATM was demonstrated to be essential for activation of AKT in response to insulin or γ‐radiation.^54^ As for TP53, the combined mutated status of TP53 and ATM was previously linked to clinical response to chemotherapies,^55^ Whereas TP53 mutants induce HER2 overexpression in cancer cells,^56^ which may constitute an anticancer resistance mechanism.^57^ To overcome cancer resistance to HER2 therapy, Fujimoto et al. suggested a combination treatment targeting PI3K/AKT/mTOR pathway in PIK3CA mutant HER2‐positive breast cancer.^58^

## 5 CONCLUSION

To our knowledge, this is the first time where the inverse relationship between ATM and HER2 was highlighted in bladder cancer. The impact of such a relationship on the prognostic outcome of patients with bladder cancer was also described. Even though our data demonstrated potential overlapping proteins between ATM‐HER2 pathways, which could contribute to bladder cancer pathogenesis, the exact molecular mechanism and biological significance of the crosstalk between HER2 and ATM still require further investigations to improve prognosis and treatment efficacy in bladder cancer. The main limitation of our study is that it was a retrospective observational study and further analyses with larger sample size are needed to investigate the relation between HER2, ATM and bladder cancer prognosis. Also, the results of the present study were based on bioinformatical analysis and must be validated further by experimental test.

## AUTHOR CONTRIBUTIONS

**Nada Albarakati:** Conceptualization (lead); data curation (lead); formal analysis (lead); methodology (lead); software (lead); writing – original draft (lead). **Alaa Al‐Shareeda:** Investigation (supporting); writing – review and editing (equal). **Majed Ramadan:** Formal analysis (supporting); methodology (equal); writing – review and editing (equal). **Batla Al‐Sowayan:** Investigation (supporting); writing – review and editing (equal). **Ola Negm:** Investigation (supporting); writing – review and editing (equal). **Taoufik Nedjadi:** Conceptualization (supporting); methodology (supporting); writing – review and editing (equal).

## CONFLICT OF INTEREST

The authors declare no conflict of interest.

## Supporting information


Figure S1–S4

Table S1–S5

## Data Availability

Data generated or analysed during this study are included in this published article and its supplementary information files. Also, both TCGA and GEO are publicly available as indicated in the materials and methods/study cohorts section.
