# Supplementary material for: Interaction between HER2 and ATM predicts poor survival in bladder cancer patients
Source: J Cell Mol Med. 2022 Sep 3;26(19):4959–73. doi: 10.1111/jcmm.17512 (PMC9549494; doi:10.1111/jcmm.17512)
Supplement: Supplementary file 1 — Figure S1–S4 [file JCMM-26-4959-s002.docx]

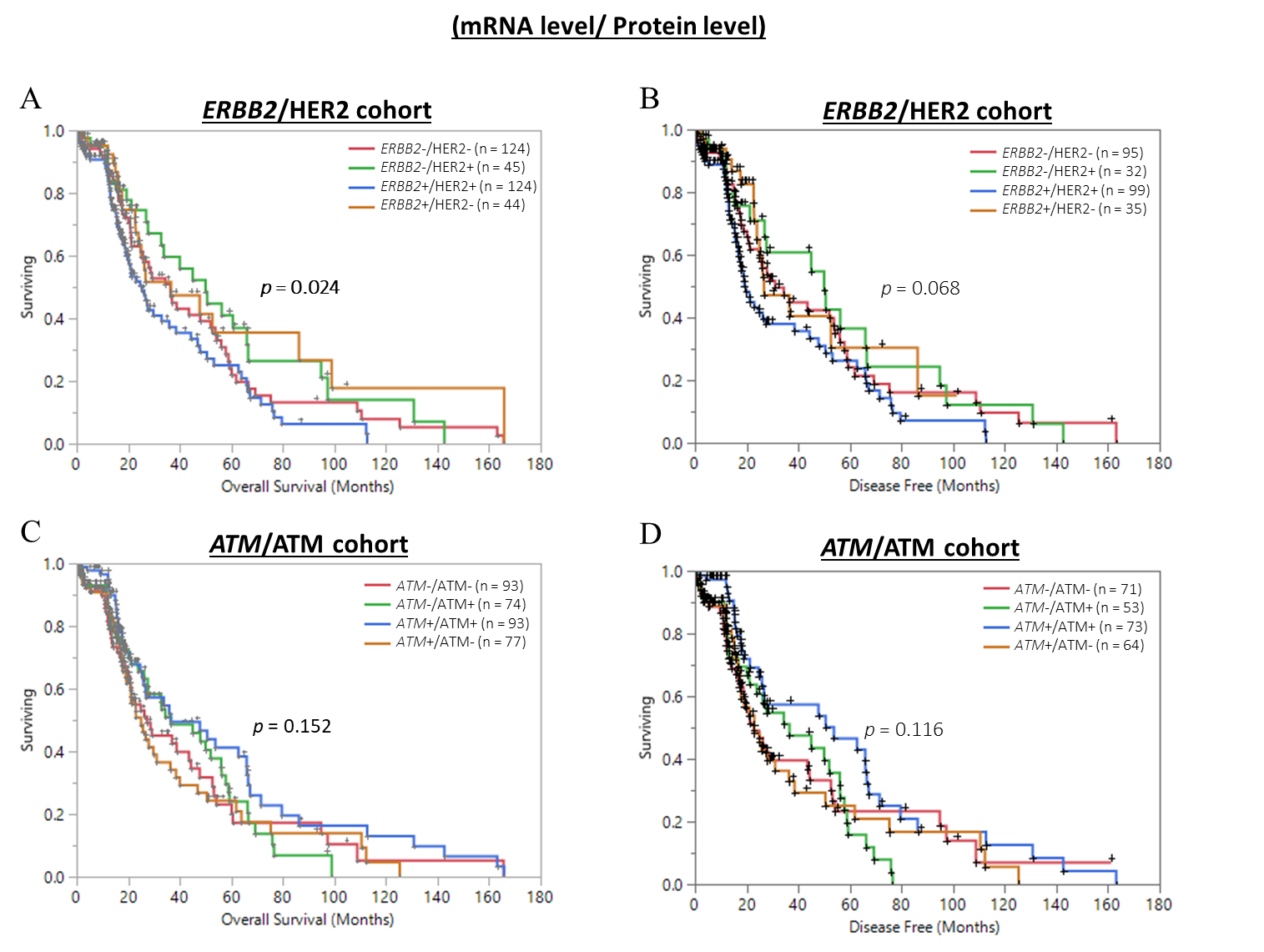
**Supporting Information**

**Supplementary Figure 1**

Kaplan-Meier analysis in bladder cancer cohort; (A) Overall survival of *ERBB2* mRNA (n=409) and HER2 different expression levels (n=344), (B) Disease free survival of *ERBB2* mRNA (n=409) and HER2 different expression levels (n=344), (C) Overall survival of *ATM* mRNA (n=409) and ATM protein different expression levels (n=344), (D) Disease free survival of *ATM* mRNA (n=409) and ATM protein different expression levels (n=344).


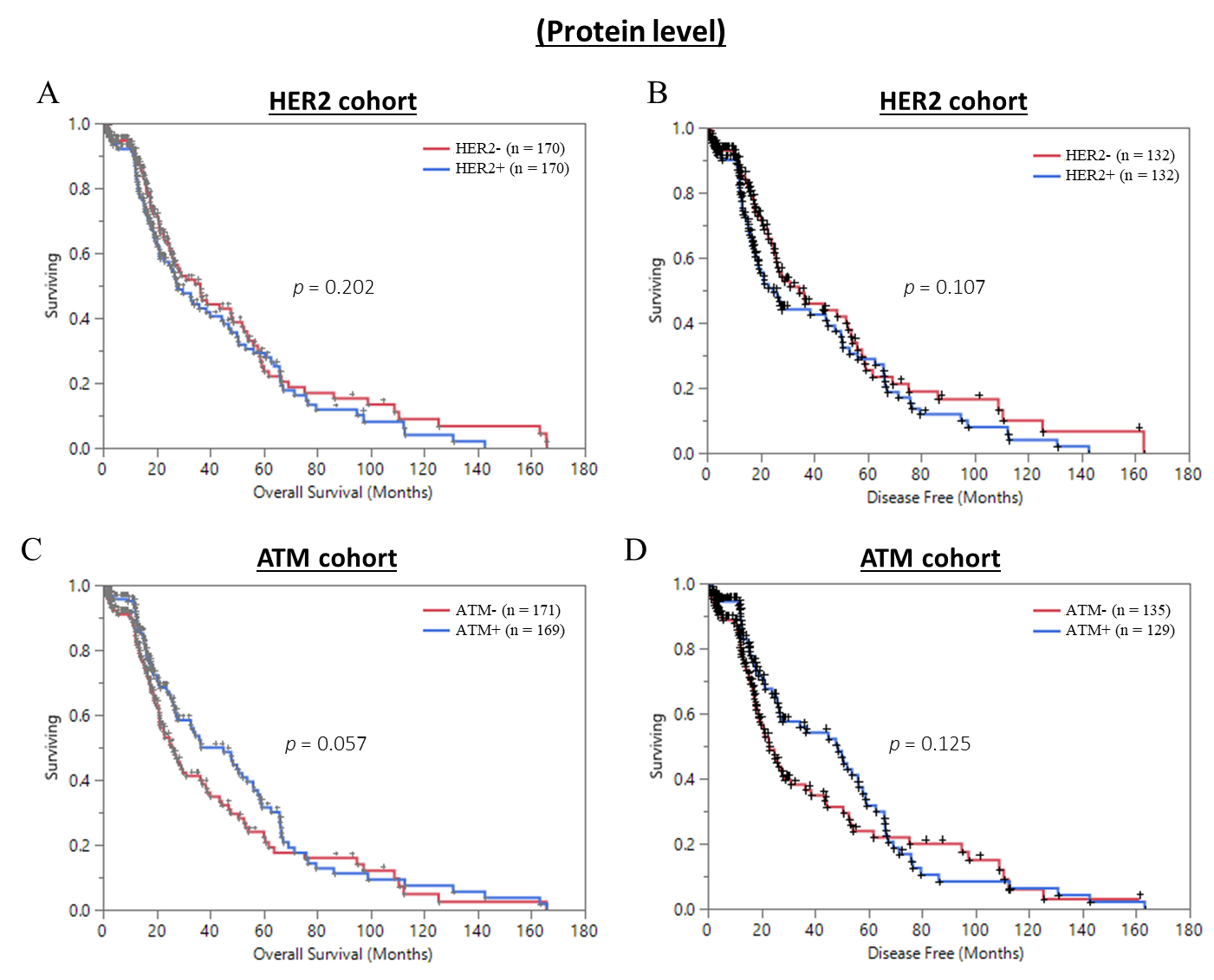


**Supplementary Figure 2**

Kaplan-Meier analysis in bladder cancer cohort; (A) Overall survival of HER2 protein expression levels (n=344), (B) Disease free survival of HER2 protein expression levels (n=344), (C) Overall survival of ATM protein expression levels (n=344), (D) Disease free survival of ATM protein expression levels (n=344).

**Supplementary Figure 3**

Kaplan-Meier analysis in bladder cancer cohort; (A) Overall survival of *ERBB2* mRNA expression levels (n=409), (B) Disease free survival of *ERBB2* mRNA expression levels (n=409), (C) Overall survival of *ATM* mRNA expression levels (n=409), (D) Disease free survival of *ATM* mRNA expression levels (n=409).


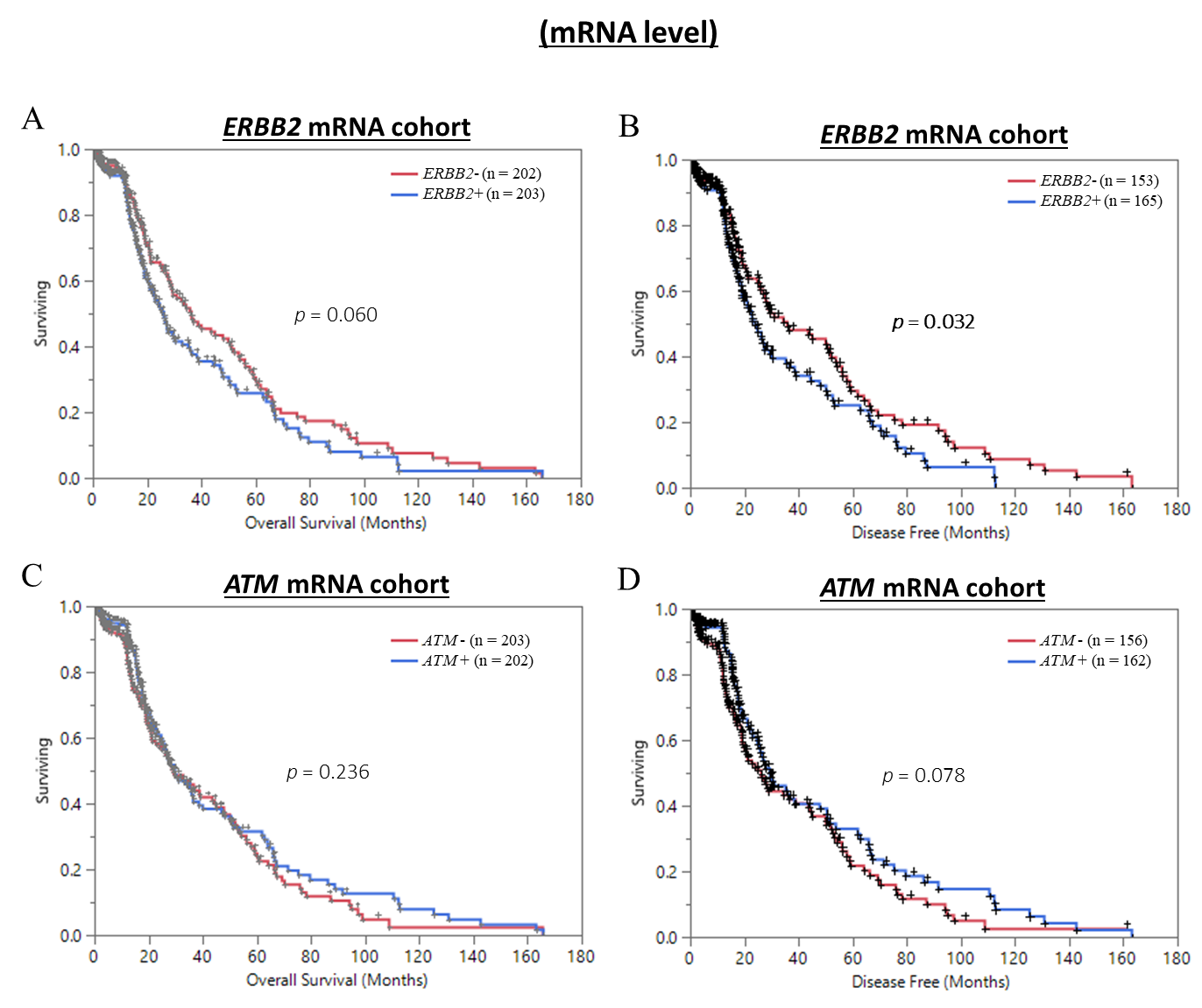


**Supplementary Figure 4**

Kaplan-Meier analysis in bladder cancer cohort; (A) Disease free survival of *ATM* mRNA expression in high *ERBB2* patients (n=205), (B) Disease free survival of *ATM* mRNA expression in low *ERBB2* mRNA patients (n=204), (C) Overall survival of *ATM* mRNA expression in high *ERBB2* mRNA patients (n=205), (D) Overall survival of *ATM* mRNA expression in low *ERBB2* patients (n=204).


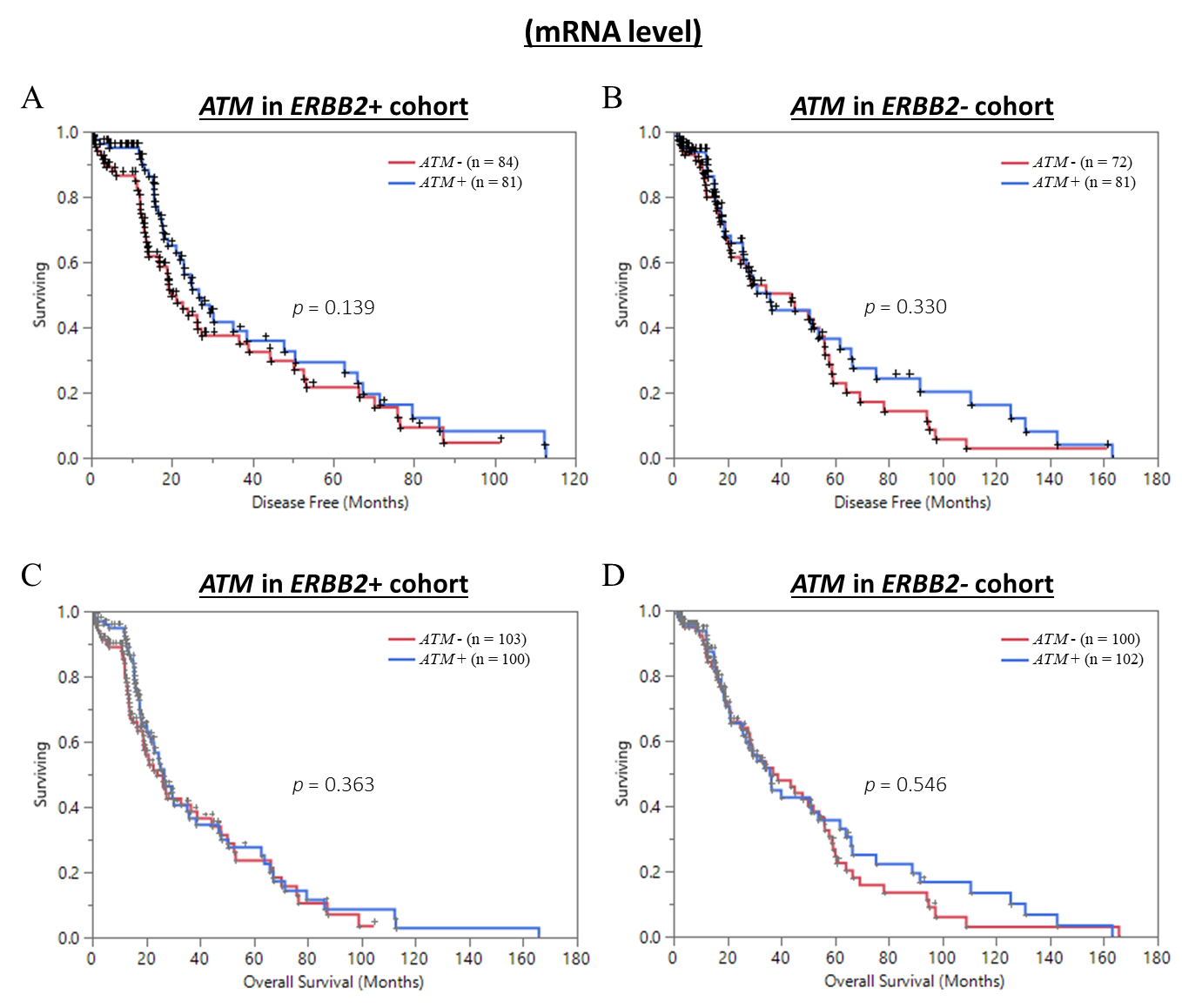


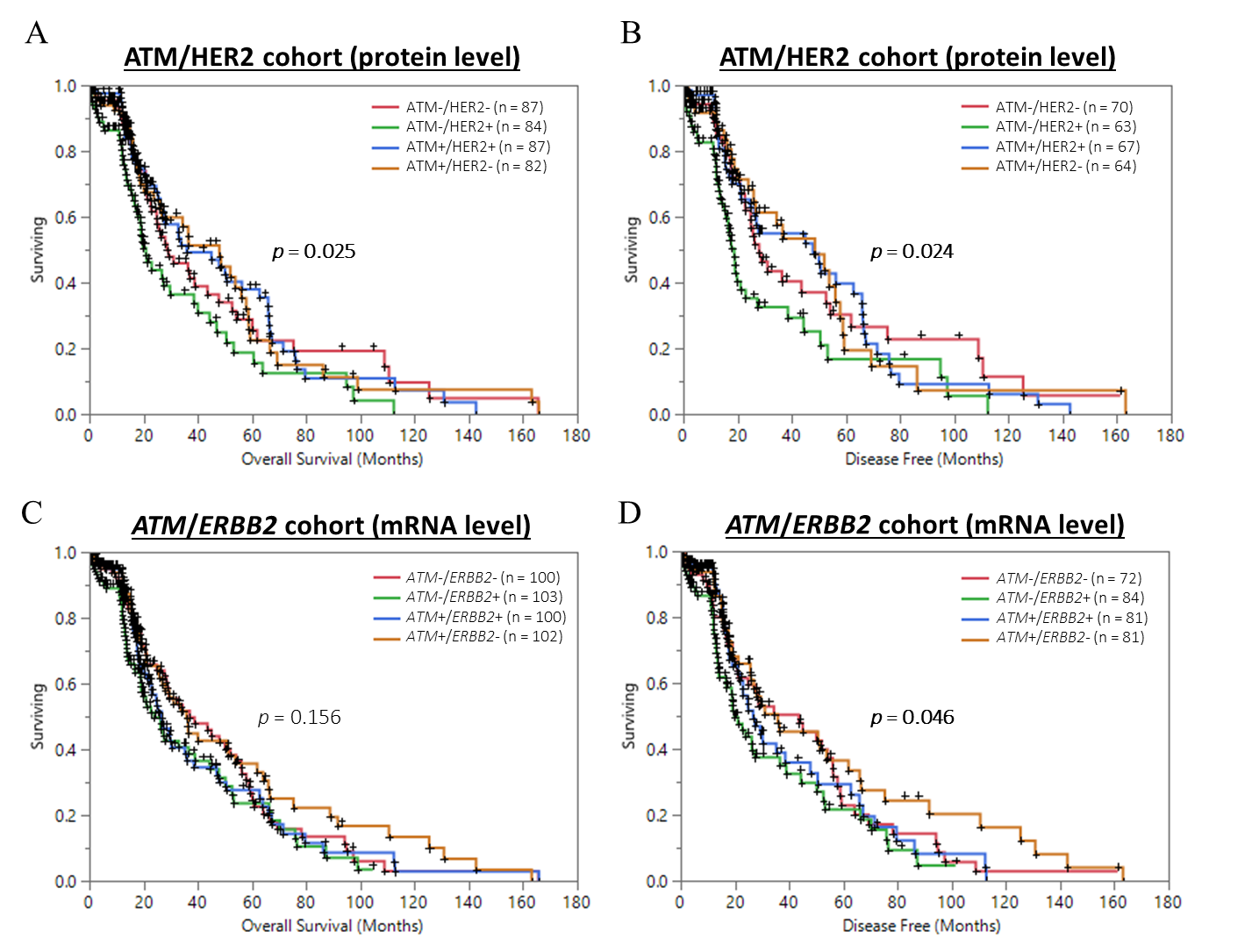
**Supplementary Figure 5**

Kaplan-Meier analysis in bladder cancer cohort of combined protein expression; (A) Overall survival of ATM/HER2 different protein expression levels (n=344), (B) Disease free survival of ATM/HER2 different protein expression levels (n=344), (C) Overall survival of *ATM/ERBB2* mRNA different expression levels (n=409), (D) Disease free survival of *ATM/ERBB2* mRNA different expression levels (n=409).
